# Supplementary material for: Deep Learning to Predict Protein Backbone Structure from High-Resolution Cryo-EM Density Maps
Source: Sci Rep. 2020 Mar 9;10:4282. doi: 10.1038/s41598-020-60598-y (PMC7063051; doi:10.1038/s41598-020-60598-y)
Supplement: Supplementary file 1 — Supplementary Information. [file 41598_2020_60598_MOESM1_ESM.pdf]

Supplementary Information

for

Deep Learning to Predict Protein Backbone Structure from High-Resolution Cryo-EM Density Maps

Dong Si[1*], Spencer A. Moritz[1], Jonas Pfab[1], Jie Hou[2,3], Renzhi Cao[4], Liguo Wang[5], Tianqi Wu[6], Jianlin Cheng[6]

[1] Division of Computing and Software Systems, University of Washington, Bothell, WA, 98011, USA
*Corresponding to Dong Si, dongsi@uw.edu

[2] Department of Computer Science, Saint Louis University, Saint Louis, MO 63103, USA
[3] Program in Bioinformatics & Computational Biology, Saint Louis University, Saint Louis, MO 63103, USA

[4] Department of Computer Science, Pacific Lutheran University, Tacoma, WA 98447, USA

[5] Department of Biological Structure, University of Washington, Seattle, WA 98185, USA

[6] Department of Electrical Engineering and Computer Science, University of Missouri, Columbia, MO 65211, USA

Table S1. Extended Table 1 - Results of simulated maps on different resolutions*.

(A) at 4Å resolution.

PDB ID	Pred. Cα	Native Cα	RMSD (Å)	% Cα in 3Å	FP%
3i2n	353	346	0.97	99.4	0.8
3n2t	342	327	1.11	99.7	0.3
3qc7	167	172	0.83	95.3	0.0
5i68	693	662	0.92	100.0	2.5
6ahv	359	345	1.01	99.7	1.9
6eyw	404	381	1.07	98.7	0.7
6g61	115	111	1.01	100.0	0.9
**Avg.**			0.99	99.0	

(B) at 5Å resolution.

PDB ID	Pred. Cα	Native Cα	RMSD (Å)	% Cα in 3Å	FP%
3i2n	350	346	0.99	99.1	0.3
3n2t	347	327	1.09	99.1	1.7
3qc7	167	172	1.03	94.8	0.0
5i68	675	662	0.91	99.5	1.3
6ahv	354	345	1.03	99.4	1.1
6eyw	384	381	0.90	98.4	0.3
6g61	111	111	0.81	100.0	0.0
**Avg.**			0.97	98.6	

*Note: There are two types of RMSD, labeled and non-labeled RMSD. The labeled RMSD considers the pair of corresponding CA positions that were labeled by the same residue number, while non-labeled doesn't. The RMSD in this table is non-labeled RMSD.

Table S2. Comparison of our method with state-of-the-art methods: Phenix, RosettaES, RosettaCM, and MAINMAST on the dataset of 43 missing fragments*.

	**Original results from Paper**					**New Run using Phenix (1.16-3549)**		**Our method**	
**Model**	**Residue_Range**	**Total_Residue**	**RosettaES**	**RosettaCM**	**MAINMAST**	**Phenix**	**Phenix_coverage**	**Final RMSD**	**coverage**
BPP1	1-111	111	2.500	48.600	3.200	0.931	0.811	1.035	0.964
BPP1	119-207	89	3.400	30.800	3.600	1.370	0.573	1.006	0.337
BPP1	207-259	53	0.900	4.200	2.100	1.636	0.038	1.378	0.226
BPP1	256-283	28	0.700	0.700	2.200	1.288	0.571	1.079	0.964
BPP1	283-327	45	2.000	30.500	6.000	1.449	0.911	1.071	0.911
FrhA	1-22	22	0.500	6.400	1.400	-	0.000	-	0.000
FrhA	136-165	30	2.100	3.200	0.900	1.339	0.900	0.933	1.000
FrhA	187-265	79	1.900	11.600	1.900	1.153	0.861	1.147	0.316
FrhA	24-29	26	0.600	0.900	0.600	-	0.000	1.301	0.833
FrhA	298-339	42	0.800	10.600	2.100	1.107	0.881	1.117	1.000
FrhA	337-358	22	0.500	0.500	1.900	1.320	0.909	0.808	1.000
FrhB	1-18	18	0.700	1.400	0.700	-	0.000	0.885	0.941
FrhB	110-143	34	0.700	3.700	1.100	1.081	0.176	1.088	0.971
FrhB	179-228	50	0.700	4.100	1.100	-	0.000	0.969	0.980
FrhB	36-66	31	1.000	1.500	1.400	0.784	0.387	1.128	0.903
FrhB	61-87	27	0.800	2.000	2.700	0.987	0.370	0.963	0.963
FrhB	87-108	22	1.000	0.800	0.700	0.661	1.000	0.908	1.000
FrhG	1-71	71	3.300	28.600	3.500	1.790	0.014	1.018	0.789
FrhG	144-172	29	0.900	1.700	1.700	2.256	0.310	0.948	0.862
FrhG	192-228	37	3.000	1.600	2.700	-	0.000	-	0.000
FrhG	73-133	61	6.600	5.900	1.600	1.824	0.311	1.097	0.967
STIV	1-163	163	16.900	45.900	17.300	1.779	0.130	1.262	0.883
STIV	161-252	92	2.300	15.900	3.200	-	0.000	1.132	0.967
STIV	252-319	68	1.800	11.200	1.300	-	0.000	1.090	0.941
T20S	13-50	38	1.600	14.600	2.100	1.529	0.132	2.144	0.474
T20S	159-220	63	1.600	37.300	4.100	1.463	0.452	2.313	0.161
T20S	43-78	36	4.300	2.100	2.200	-	0.000	2.126	0.472
T20S	88-166	79	1.100	5.900	6.000	1.242	0.430	2.173	0.266
TMV	1-11	11	0.700	0.600	0.700	0.523	0.636	0.834	0.909

TMV	44-78	35	0.800	0.800	1.100	0.913	0.829	0.949	1.000
TMV	78-106	29	3.200	1.500	1.000	0.992	0.621	1.112	0.241
TRPV1	1-45	45	6.100	3.000	2.600	1.612	0.644	1.918	0.044
TRPV1	111-134	24	1.600	5.600	1.800	1.399	0.895	1.262	0.947
TRPV1	128-183	56	4.000	4.800	1.600	0.766	0.964	1.221	0.964
TRPV1	205-226	22	3.400	0.700	1.500	0.938	0.955	0.956	1.000
TRPV1	226-310	85	7.000	3.500	3.000	0.869	0.929	1.097	0.953
TRPV1	66-110	45	1.400	1.700	2.900	0.588	0.622	1.248	0.556
VP6	1-81	81	2.600	43.300	3.900	1.144	0.049	1.286	0.975
VP6	115-245	131	4.200	38.500	11.000	2.050	0.145	1.554	0.832
VP6	243-269	27	2.900	2.000	1.800	-	0.000	1.587	1.000
VP6	266-299	34	0.700	8.500	1.100	-	0.000	1.604	0.971
VP6	300-350	51	0.600	3.600	2.300	-	0.000	1.546	0.922
VP6	349-372	24	1.100	1.000	1.000	-	0.000	1.867	0.250
VP6	87-118	32	0.600	1.600	1.400	1.339	0.969	1.316	1.000
**avg.**			**2.389**	**10.293**	**2.681**	**1.254 (exclude 0 coverage)**	**0.419**	**1.273 (exclude 0 coverage)**	**0.742**

*Note: The results of MAINMAST and Rosetta are from MAINMAST paper (Terashi, et al. *Nature communications*, 2018). The labeled RMSD is used for MAINMAST and Rosetta. We use "-" to fill the fields that are not covered by the prediction. Non-labeled RMSD and matching percentage are calculated based on Phenix.chain_comparison https://www.phenix-online.org/documentation/reference/chain_comparison.html).

Table S3. Comparison of Phenix, Rosetta do-novo, MAINMAST and our method on the dataset of 30 experimental maps*.

	**MAINMAST**		**Rosetta de-novo**		**Phenix**		**Our method**	
**EMDB ID**	**Matching Percentage for MAINMAST**	**RMSD for MAINMAST**	**Matching Percentage for Rosetta**	**RMSD for Rosetta**	**Matching Percentage for Phenix**	**RMSD for Phenix**	**Matching Percentage for our method**	**RMSD for our method**
1461A	0.860	30.600	0.780	17.900	0.018	1.117	0.917	1.336
2364A	0.670	34.700	-	-	0.021	1.816	0.586	1.941
2513A	0.960	3.800	0.900	9.900	0.128	1.143	0.982	1.030
2513B	0.960	4.300	0.680	30.500	0.031	1.758	0.947	1.166
2513C	0.930	4.400	0.850	8.800	0.065	1.855	0.971	1.039
2850A	0.790	23.500	0.840	14.700	0.042	1.795	0.733	1.410
2867B	0.900	9.300	0.830	10.600	0.032	1.340	0.859	1.503
3063C	0.720	34.000	-	-	0.025	1.733	0.809	1.532
3073A	0.880	40.400	0.820	14.900	0.030	1.288	0.861	1.268
3231K	0.890	15.800	0.830	20.800	0.081	1.774	0.231	1.644
3246A	0.720	19.600	0.510	24.700	0.237	1.401	0.463	2.205
3246B	0.840	17.400	0.430	49.100	0.032	1.965	0.942	1.272
5155A	0.830	41.500	0.230	96.600	0.028	1.763	0.915	1.346
5185A	1.000	2.700	0.990	1.200	0.000	0.000	0.917	1.281
5376D	0.830	25.700	0.750	18.700	0.014	1.171	0.808	1.445
5495A	0.950	9.600	0.330	67.700	0.000	0.000	0.967	1.136
5584A	0.880	33.400	0.520	57.000	0.094	2.164	0.936	1.165
5764A	0.890	36.100	0.660	30.700	0.026	1.263	0.948	1.168
5778A	0.900	6.300	0.890	7.200	0.013	1.758	0.757	1.243
5925A	0.960	3.600	0.990	1.000	0.545	1.262	0.979	1.221
6219A	0.860	25.600	0.760	25.500	0.022	0.608	0.864	1.545
6272A	0.960	17.600	0.780	19.700	0.043	0.861	0.992	0.801
6374D	0.990	1.700	0.850	8.100	0.025	1.262	0.979	0.952
6478A	0.980	2.600	0.490	42.000	0.084	1.896	0.971	1.173
6551A	0.930	4.400	0.870	12.300	0.090	0.897	0.954	1.167
6555A	0.970	2.400	0.480	30.400	0.102	1.964	0.958	0.971
8011D	0.870	11.300	0.200	45.700	0.006	1.770	0.854	1.307
8015A	0.990	2.200	0.350	64.300	0.082	1.992	0.993	0.928
8116A	0.760	49.800	0.880	10.700	0.035	1.715	0.844	1.325
**Avg.**	**0.885**	**17.734**	**0.685**	**27.433**	**0.067**	**1.425**	**0.860**	**1.294**

*Note: The data of MAINMAST and Rosetta are from MAINMAST paper (Terashi, et al. *Nature communications*, 2018). The labeled RMSD is used for MAINMAST and Rosetta. We use "-" to fill the fields that are not covered by the prediction. Non-labeled RMSD and matching percentage are calculated based on Phenix.chain_comparison https://www.phenix-online.org/documentation/reference/chain_comparison.html).

Table S4. Comparison of our method with state-of-the-art methods: Phenix, Rosetta-denovo, and MAINMAST using 3Å resolution simulated density maps*.

PDB	T1	TM-score				GDT-TS				RMSD				Coverage of Residues in predicted structure			
		T2	T3	T4	T5	T2	T3	T4	T5	T2	T3	T4	T5	T2	T3	T4	T5
3i2n	357/345	0.345	0.269	0.217	-	0.205	0.251	0.123	-	16.603	18.666	20.595	-	80.11%	43.98%	69.19%	-
3n2t	348/327	0.818	0.713	0.913	0.900	0.573	0.676	0.848	0.766	3.397	2.111	2.653	3.239	100.00%	70.69%	93.10%	93.10%
3qc7	179/164	0.522	0.809	0.711	0.255	0.384	0.803	0.657	0.191	5.030	1.281	3.913	13.354	100.00%	78.21%	88.27%	88.27%
5i68	663/662	0.726	0.493	0.335	0.364	0.325	0.455	0.263	0.258	5.961	8.027	24.618	27.810	100.00%	54.45%	66.82%	99.85%
6ahv	363/345	0.846	0.558	0.795	0.780	0.625	0.527	0.670	0.596	3.125	2.914	5.003	5.497	100.00%	57.30%	96.14%	95.04%
6eyw	427/381	0.265	0.848	0.815	0.185	0.217	0.833	0.764	0.081	17.335	1.340	6.994	22.894	100.00%	79.39%	85.48%	100.00%
6g61	133/111	0.234	0.931	0.251	0.553	0.201	0.941	0.246	0.527	12.657	1.968	0.225	7.587	100.00%	81.20%	82.71%	100.00%
Average		0.537	0.660	0.577	0.506	0.361	0.641	0.510	0.403	9.158	5.187	9.143	13.397	97.16%	66.46%	83.10%	96.04%

*Note - T1: Residues in sequence/native; T2: Our method; T3: Rosetta de-novo; T4: Phenix; T5: MAINMAST. The labeled RMSD is used for MAINMAST and Rosetta.

Table S5. Comparison of our method with state-of-the-art methods: Phenix, Rosetta-denovo, and MAINMAST in three experimental maps*.

PDB	Method	Fragments	TM-score	GDT-TS	RMSD
6272	Our method	Full_length (1-397)	0.932	0.809	2.133
	Rosetta-Denovo	Full_length (1-397)	0.581	0.534	11.432
		Fragment 1 (1-47)	0.844	0.894	1.802
		Fragment 2 (67-158)	0.802	0.81	1.766
		Fragment 3 (216-310)	0.532	0.513	7.113
		Fragment 4 (338-397)	0.749	0.796	0.675
	Phenix	Full_length (1-397)	0.6144	0.5164	16.914
		Fragment 1 (1-144)	0.77	0.748	14.038
		Fragment 2 (149-266)	0.175	0.146	0.095
		Fragment 3 (273-396)	0.789	0.768	2.825
	MAINMAST	Full_length (1-397)	0.528	0.334	14.79
5778	Our method	Full_length (1-586)	0.452	0.286	24.342
		Fragment 1 (253-581)	0.702	0.509	6.497
		Fragment 2 (167-245)	0.402	0.459	5.51
	Rosetta-Denovo	Full_length (1-586)	0.168	0.161	0.142
		Fragment 1 (464-574)	0.848	0.842	0.964
	Phenix	Full_length (1-586)	0.219	0.173	15.186
		Fragment 1 (87-117)	0.359	0.444	8.473
		Fragment 2 (354-423)	0.461	0.577	15.214
		Fragment 3 (470-493)	0.587	0.875	1.622
		Fragment 4 (519-579)	0.667	0.742	2.448
	MAINMAST	Full_length (1-586)	0.354	0.129	20.636

8410	Our method	Full_length (1-890)	0.475	0.251	48.191
		Fragment 1 (78-560)	0.786	0.46	4.455
	Rosetta-Denovo	Full_length (1-890)	0.076	0.072	9.66
		Fragment 1 (284-300)	0.579	0.927	1.663
		Fragment 2 (395-406)	0.377	0.708	7.097
		Fragment 3 (479-499)	0.468	0.976	0.637
		Fragment 4 (742-755)	0.573	0.946	0.91
		Fragment 5 (835-850)	0.387	0.906	1.045
	Phenix	Full_length (1-890)	0.184	0.093	51.833
		Fragment 1 (1-221)	0.239	0.192	33.084
		Fragment 2 (366-462)	0.308	0.312	16.039
		Fragment 3 (754-823)	0.176	0.225	12.38
	MAINMAST	Full_length (1-890)	0.119	0.025	41.171

*Note: The labeled RMSD is used for MAINMAST and Rosetta.

Impact of Manual Threshold Selection

As mentioned in the Methods section, voxels below a certain threshold are zeroed out in a pre-processing step. This aims to reduce noise and allows the CNN to focus on high intensity voxels. However, this threshold has to be selected manually, which is a non-trivial task prone to error. Therefore, we examine how sensitive the accuracy of the backbone predictions is to variations in the selected threshold. To do so, we predict the backbone structure of three density maps using varying thresholds in a range of -100% to +100% of the manually selected threshold (see Table S6). As we can see the prediction accuracy is affected by the selected threshold, however, only to a limited extend. Particularly within the range of -25% to +25% of the manually selected threshold the accuracy variations are only minor.

Table S6. Prediction results of three density maps for thresholds varying between -100% to +100% of the manually selected threshold

**Threshold**	**EMD 8482**			**EMD 8515**			**EMD 8642**		
	**Pred. Cα**	**RMSD**	**% Cα in 3Å**	**Pred. Cα**	**RMSD**	**% Cα in 3Å**	**Pred. Cα**	**RMSD**	**% Cα in 3Å**
-100%	5334	1.74	76.4	5225	1.34	75.2	4187	1.50	74.7
-75%	2770	1.63	77.1	4815	1.25	76.6	2339	1.49	74.6
-50%	1677	1.67	74.6	4505	1.23	75.4	1198	1.54	74.6
-25%	1229	1.60	71.6	4042	1.26	76.6	819	1.39	84.3
0%	1098	1.60	70.2	3594	1.12	76.5	731	1.36	84.8
+25%	1021	1.61	70.4	3201	1.10	76.9	657	1.31	83.4
+50%	914	1.63	66.2	2962	1.07	74.9	577	1.36	76.5
+75%	833	1.64	62.0	2824	1.10	74.4	500	1.45	67.4
+100%	698	1.70	55.5	2736	1.13	72.3	429	1.48	58.2

Runtime of Predictions

In the following chart we can see the runtime of the predictions relative to how many backbone Cα atoms were predicted. The time is measured for the end-to-end processing time. However, the vast majority of the time is spent on the global Tabu-Search and chain tracing algorithm in post-processing step. The C-CNN voxel prediction step only takes less than a minute, even for the large maps. The format of the elapsed time is 'hh:mm:ss'.

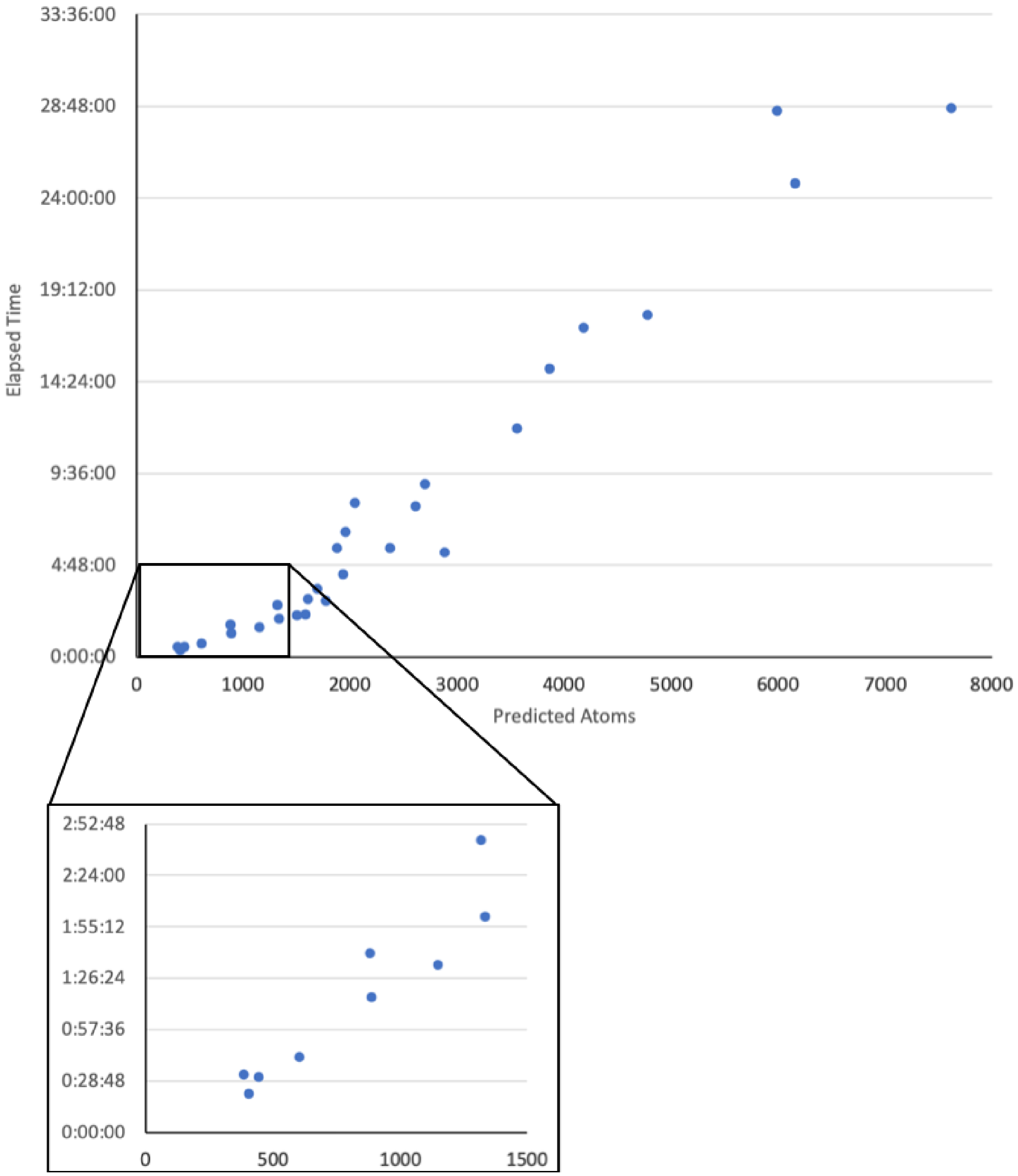

Tabu-Search Algorithm

Below is the Python pseudo-code for our tabu-search algorithm. The confidence map corresponds to the Ca confidence map that is returned from the C-CNN. The result of the method is a list of traces and each trace is in itself a list of atoms. The confidence_map[atom] value expresses the confidence value at the location of an atom.

```
def tabu_search(confidence_map):
	traces = []
	while next_atom(confidence, traces) is not None:
		# Atom at which trace is continued
		atom = next_atom(confidence, traces)

		# Find next best atom to continue trace
		neighbor_atom = find_nearest_atom(atom, confidence_map)
		if neighbor_atom is not None:
			# Append neighbor atom to corresponding trace
			append_to_trace(traces, atom, neighbor_atom)

	return traces

def next_atom(confidence_map, traces):
	if len(traces) > 0:
		possible_atoms = []
		for trace in traces:
			# Only the first and last atom of each trace can be the next atom
			possible_atoms += [trace[0], traces[-1]]

		# Return atom with the highest value in the Ca confidence map
		return max(possible_atoms, key=lambda atom: confidence_map[atom])
	else:
		# Atom at global max in Ca confidence map
		atom = global_max(confidence_map)

		# Return atom if its confidence value is higher than some threshold
		return atom if confidence_map[atom] >= 0.5 else None
```

Preprocessing

The experimental cryo-EM maps were segmented using UCSF Chimera's "zone" tool if the deposited structure is only fitted in part of the density map, as described in the Rosetta and MAINMAST paper. The first step of the preprocessing was to use hideDust in Chimera to remove any dust from the density map (optional step). This was done with setting the contour levels to low and high to visualize the dust and then using Chimera's command hideDust to pick a size that removed any outliers that were making noise. The hide dust sizes and the low or high level contour levels could be inputted as a json file to the program.

Running Rosetta-Denovo:

Rosetta (rosetta_bin_linux_2018.33.60351_bundle) was used. We followed the tutorial released on http://dimaiolab.ipd.uw.edu/software/. Almost all the parameters used were as described in the tutorial, but some specific parameters were taken from the following paper: Wang, R. Y. R., Kudryashev, M., Li, X., Egelman, E. H., Basler, M., Cheng, Y., Baker, D., & DiMaio, F. (2015). De novo protein structure determination from near-atomic-resolution cryo-EM maps. Nature Methods, 12(4), 335-338.

First, fragment structures for the query protein were generated on the Robetta website: http://robetta.bakerlab.org/fragmentqueue.jsp

1. Local Fragment search in an input EM map using denovo_density.

This procedure searches the density map for each sequence-predicted backbone fragment generated in the previous step.

```
$ROSETTA3/source/bin/denovo_density.static.linuxgccrelease \
-in::file::fasta ./target.seq \
-fragfile ./aaabini09_05.200_v1_3 \
-startmodel ./start_model.pdb \
-mapfile ./MAP.mrc \
-n_to_search 1500 -n_filtered 3000 -n_output 100 \
-bw 16 \
-atom_mask_min 2 \
-atom_mask 3 \
-clust_radius 2 \
-clust_oversample 4 \
-movestep 1 \
-delR 2 \
-frag_dens 0.8 \
-ncyc 3 \
-min_bb false \
-pos $1 \
-out:file:silent round$2/fragment.$1.silent
```

2. Placed fragment scoring using denovo_density:

This step score the placement of fragments for compatibility to the EM map.

```
$ROSETTA3/sourcebin/denovo_density.static.linuxgccrelease \
-mode score \
-in::file::silent round1/fragment*silent \
-scorefile round1/scores1 \
-n_matches 50
```

3. Monte Carlo fragment assembly using denovo_density:

This step generates “maximally consistent” fragment assembly in the map.

```
$ROSETTA3/source/bin/denovo_density.static.linuxgccrelease \
-mode assemble \
-nstruct 5 \
-in::file::silent round1/fragment*silent \
-scorefile round1/scores1 \
```

```
-assembly_weights 4 20 6 \
-null_weight -150 \
-out:file:silent round1/assembled.$1 \
-scale_cycles 1 \
-mute core
```

4. Consensus assignment using denovo_density:
This step is to identify the consensus assignment from the lower-scoring Monte Carlo Trajectories.

```
$ROSETTA3/source/bin/denovo_density.static.linuxgccrelease \
-mode consensus \
-in::file::silent round1/assembled.*silent \
-consensus_frac 1.0 -energy_cut 0.05 \
-mute core
```

If the assigned backbone residues are less than 70% of the target protein or the coverage is not converged, we iterate the four (1-4) steps.

Running Phenix:

We followed the tutorials of Phenix(1.16-3549) released on https://www.phenix-online.org/documentation/reference/map_to_model.html. The default parameters are used, and the command is as follows:

```
phenix.auto_sharpen 6272.mrc resolution=2.6 sharpened_map_file=density.ccp4
```

```
phenix.map_to_model density.ccp4 seq_file=6272.fasta resolution=2.6 nproc=10 pdb_out=result.pdb find_symmetry=True thoroughness=quick
```

Running MAINMAST:

We followed the tutorials of MAINMAST released on http://kiharalab.org/mainmast/Tutorials.html. The following command is used:

For segmented map:

```
$MAINMAST/MAINMAST -m target.situs -filter 0.3 -Rlocal 10 > target_path.pdb
```

For simulated map:

```
$MAINMAST/MAINMAST -m target.situs -filter 0.3 -Dkeep 1.0 -Ntb 10 -Rlocal 5 -Nlocal 50 -Nround 50 > target_path.pdb
```

For threading:

```
$MAINMAST/ThreadCA -i target_path.pdb -a $MAINMAST/20AA.param -spd target.spd3 -fw 1.3 -Ab 3.3 -Wb 0.9 > prediction.pdb
```
